# PAMPAS: A PsychoAcoustical Method for the Perceptual Analysis of multidimensional Sonification

**DOI:** 10.3389/fnins.2022.930944

**Published:** 2022-10-06

**Authors:** Tim Ziemer, Holger Schultheis

**Affiliations:** Bremen Spatial Cognition Center, University of Bremen, Bremen, Germany

**Keywords:** sonification evaluation, psychoacoustics, just noticeable difference, difference limen, discrimination threshold, comparison of sonification designs, maximum likelihood procedure, auditory display

## Abstract

The sonification of data to communicate information to a user is a relatively new approach that established itself around the 1990s. To date, many researchers have designed their individual sonification from scratch. There are no standards in sonification design and evaluation. But researchers and practitioners have formulated several requirements and established several methods. There is a wide consensus that psychoacocustics could play an important role in the sonification design and evaluation phase. But this requires a) an adaption of psychoacoustic methods to the signal types of sonification and b) a preparation of the sonification for the psychoacoustic experiment procedure. In this method paper, we present a PsychoAcoustical Method for the Perceptual Analysis of multidimensional Sonification (PAMPAS) dedicated to the researchers of sonification. A well-defined and well-established, efficient, reliable, and replicable just noticeable difference (JND) experiment using the maximum likelihood procedure (MLP) serves as the basis to achieve perceptual linearity of parameter mapping during the sonification design stage and to identify and quantify perceptual effects during the sonification evaluation stage, namely the perceptual resolution, hysteresis effects and perceptual interferences. The experiment results are scores from standardized data space and a standardized procedure. These scores can serve to compare multiple sonification designs of a single researcher or even among different research groups. This method can supplement other sonification designs and evaluation methods from a perceptual viewpoint.

## 1. Introduction

Sonification is the systematic conversion from data to sound thus the aspects of the data become unambiguously understandable for a user (Hermann, 2008, 2021; Scaletti, 2018). Even though antecedents can be found throughout all periods of history, *sonification* as a term and as a dedicated field of research established itself around the early 1990s (Worrall, 2019, chap. 1).

Sonification research is interdisciplinary by nature, as it deals with acoustics and audio signal processing, sound design and composition, human-machine interaction, cognition and human factors, auditory perception, and many more disciplines. A strength of this interdisciplinarity is that researchers bring in research questions and methods from the viewpoint of their discipline. But at the same time, it is unlikely that a single sonification researcher, or even a research group, can cover all relevant disciplines. This becomes a drawback if questions and methods from a discipline get neglected because they are unknown or not well understood. This is sometimes true from the psychoacoustic viewpoint on sonification design and evaluation. Even though some sonification researchers pointed out the potential of psychoacoustic methods (Brewster, 2003; Ferguson et al., 2006; Bovermann et al., 2011; Walker and Nees, 2011), they were not often applied during the design and evaluation stage of sonification. Other researchers raised doubts about the appropriateness of psychoacoustic methods (Smith et al., 1994; Anderson and Sanderson, 2009; Vogt, 2011).

This methods paper contributes to the discussion of the appropriateness of psychoacoustic methods in sonification research, to clear up prejudices and misconceptions, and to provide sonification researchers with an applicable, psychoacoustic method for the design and evaluation of multidimensional sonification.

### 1.1. Problem statement

**Figure 1:**
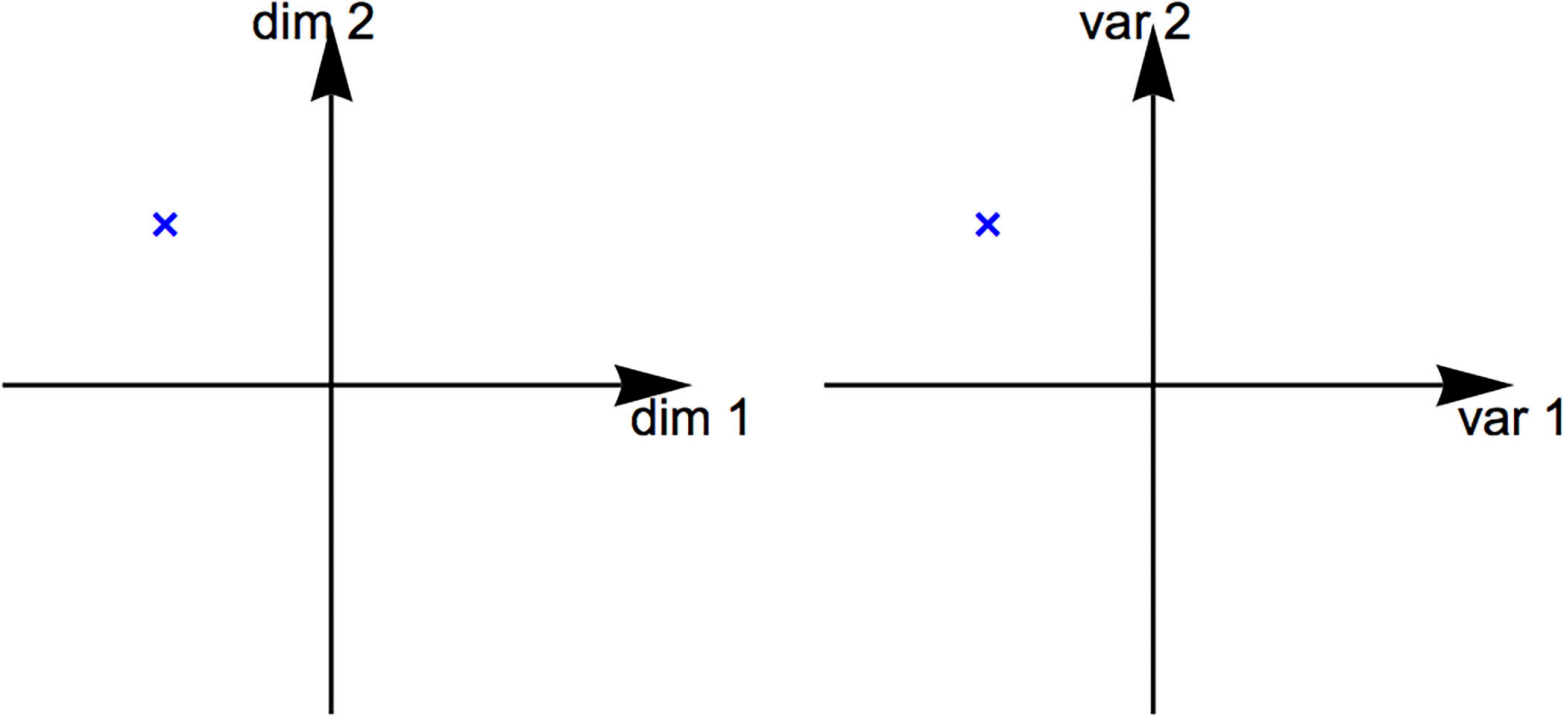
Multidimensional space (left) and multivariate space (right). For auditory and visual data representation, dimensions and variables can sometimes be treated the same.

Sometimes sonified data is multidimensional. Examples include an abstract phase space (Hermann, 2018), real spatial locations (Lokki and Gröhn, 2005; Ziemer and Schultheis, 2019a; Ziemer et al., 2020), and angles (Greindl et al., 2020; Asendorf et al., 2021). A point in a multidimensional space has several coordinates. Sometimes, data is multivariate. Examples include vital functions of patients, such as pulse frequency and blood oxygen concentration (Yeung, 1980; Fitch and Kramer, 1993; Watson and Sanderson, 2004; Ziemer et al., 2020), geopolitical data, such as crime rate and unemployment rate (Olivetti Belardinelli et al., 2009), or pH and chlorine level in water (Ziemer et al., 2020). A point in a multivariate space has multiple variables or attributes. Dimensions and variables are not the same. But sometimes they are considered the same, for example, the case of data presentation, as illustrated in Figure 1. A treatise of multiple dimensions and variables in data presentation can be found in Munzner (2014). In this study, we refer to both cases as being *multidimensional*. Furthermore, we refer to the sonification of multidimensional data as *multidimensional sonification*.

In the sonification literature, there is consensus that *auditory perception of each sonified dimension* needs to fulfill 4 requirements:

1. A sonification should be perceived as linear (Barrass, 1997, p. 115; Hermann, 2002, p. 39; Worrall, 2019, p. 42; Ziemer and Schultheis, 2020; Ziemer et al., 2020). In a linear system, the output is directly proportional to the input thus the doubling of an input value doubles the output value. Linearity is necessary to transfer data relations and proportions from the data domain to the auditory domain. In psychophysics, a logarithmic transform of the physical input sometimes produces a fairly linear sensational output (Fechner, 1860, pp. 134ff; Schneider, 2018a). For example, a constant *frequency ratio* tends to sound like a fairly constant *pitch interval*. According to the psychoacoustic literature (Fechner, 1860, p. 60; Schneider, 2018a), a linear sensory scale is achieved when the just noticeable difference (JND) is constant throughout the scale.
2. A sonification should exhibit a high perceptual resolution (Yeung, 1980; Barrass, 1997, chap. 7.7.5; Hermann, 2002, pp. 34 & 40; Brewster, 2003; Vickers, 2006; Ziemer and Schultheis, 2019b, 2020; Ziemer et al., 2020). The JND is an adequate measure of perceptual resolution, which is acquired in psychoacoustic experiments. Here, all but one physical parameter tend to be kept constant, so the JND of the remaining parameter can be measured. For example, the JND in the frequency of pure tones evolves from 1% at low frequencies to 0.3% at midrange frequencies to 1% at high frequencies (Scheminzky, 1943, p. 136; Ziemer, 2020, chap. 4). The JND in the amplitude of a pure tone is 0.3 to 1.4 dB, depending on reference amplitude and frequency (Ziemer, 2020, pp. 74f).
3. Sonifications should exhibit preferably little hysteresis effects (Neuhoff, 1998; Neuhoff and Kramer, 2000; Martens, 2002; Feron et al., 2009). Hysteresis effects are well-known in the field of psychoacoustics and even exist in fundamental aspects of sound perception, such as pitch (Stevens and Volkmann, 1940; Greenwood, 1997; Chambers and Pressnitzer, 2014) and loudness (Canévet and Scharf, 1990). Hysteresis is the effect that the distance from data point **a** to **b** may be perceived differently than the distance from **b** to **a**. This means the difference not only depends on the interval but also on where you are coming from. In sonification research, hysteresis is typically neither quantified nor qualitatively discussed.
4. Each sonified dimension should exhibit little perceptual interference with other dimensions (Yeung, 1980; Barrass, 1997, p. 16; Barrass and Kramer, 1999; Neuhoff and Kramer, 2000; Hermann, 2002, p. 34; Neuhoff et al., 2002; Brewster, 2003; Ferguson et al., 2006; Anderson and Sanderson, 2009; Worrall, 2014; Ziemer et al., 2018, 2020; Neuhoff, 2019; Ziemer and Schultheis, 2019b; Worrall, 2019, chap. 2.2.2.2; Ziemer and Schultheis, 2019c, 2020). This means that changes along one data dimension sound like changes along two or more data dimensions, or that the absolute magnitude of data dimension one affects the resolution of data dimension two. Perceptual interference can “obscure data relations and confuse the listener” (Worrall, 2019, chap. 2.2.2.2). In the field of psychoacoustics, it is accepted that all physical aspects of sound can affect practically all aspects of sound perception. For example, even though amplitude and frequency of a pure tone are physically orthogonal, they both interfere perceptually, as both can affect the sensation of, e.g., loudness and pitch (Zwicker and Fastl, 1999, chaps. 5.1.2 and 8.1; Schneider, 2018b). In the psychoacoustic literature, fundamental aspects of sound sensation like loudness, roughness, sharpness, and tonalness are considered largely independent from one another (Aures, 1985a; Zwicker and Fastl, 1999, chap. 9), albeit all of them can be influenced by very many physical sound parameters to some extent. Even though the psychoacoustic literature tends to concentrate on the forward problem, psychoacoustic methods and models can provide aid for a psychoacoustic sonification design.

Unfortunately, the literature neither provides any guidelines to design a multidimensional sonification that fulfills these requirements nor a method to quantify if and to what extent a multidimensional sonification meets these requirements.

In this method paper, we, therefore, propose a **P**sycho**A**coustic **M**ethod for the **P**erceptual **A**nalysis of multidimensional **S**onification (PAMPAS). PAMPAS includes the preparation of the multidimensional sonification for psychoacoustic testing and some established and some new ways of interpreting the experimental results dedicated to answer the question of sonification researchers. In particular, it serves as an aid to

1. achieve perceptual linearity during the sonification design stage and as a tool
2. assess the perceptual resolution
3. assess hysteresis effects
4. assess perceptual interference during the sonification evaluation stage
5. and enable comparison between sonification designs of various studies.

The remainder of this method paper is structured as follows: we start with the common practice in sonification design and evaluation in Section 2. The section summarizes the common practice and discussions about the potentials and limitations of psychoacoustic methods for sonification research. Section 3 lists the materials and equipment needed to carry out the proposed psychoacoustic experiment. Section 4 describes the proposed psychoacoustic method that is supposed to overcome the current limitations of psychoacoustic procedures by being optimized for the signal types and the requirements to be expected in multidimensional sonification. Section 5 demonstrates how to extract and report the expected experiment results. Section 6 concludes the work, discusses its strengths and weaknesses, and gives an outlook on further psychoacoustic methods that could be adapted to become utilizable for sonification research.

## 2. Common practice in multidimensional sonification

In the field of sonification research, many works highlight that there is a need for, but a lack of, comprehensive sonification design guidelines (Nees and Walker, 2009; Ibrahim et al., 2011; Supper, 2012). In contrast to that, many sonification evaluation methods, especially listening tests (Seiça et al., 2020), have proven their value in several studies.

### 2.1. Conventional sonification design and evaluation

The chapter on Auditory Display Evaluation in The Sonification Handbook suggested that experts should use their own introspection and intuition, especially in the early stage of sonification design (Bonebright and Flowers, 2011, p. 112; Vogt, 2011) agreed to the necessity for sonification researchers to evaluate their initial sonification designs subjectively. After this early sonification design stage, “a variety of methods including both laboratory components and ecologically valid testing” (Bonebright and Flowers, 2011, p. 111) is recommended.

Worrall (2014) discusses the mapping problem and highlights the need for heuristic testing to approach multidimensional sonification. Neuhoff et al. (2002) underline the need for interdisciplinary research for developing and evaluating auditory displays. Typically, sonification evaluations with potential end-users are presented in the sonification literature (Seiça et al., 2020), rather than initial self-tests (Ibrahim et al., 2011). To evaluate their sonification design, many researchers let users solve a task with auditory, visual, and audiovisual guidance (Lokki and Gröhn, 2005; Black et al., 2017; Ziemer and Schultheis, 2019a), others compare multiple sonification designs for a specific task (Walker and Lindsay, 2006; Albrecht et al., 2016; Komatsu and Yamada, 2016; Parseihian et al., 2016). In navigation studies, researchers evaluate for example completion time (Lokki and Gröhn, 2005; Walker and Lindsay, 2006; Hansen et al., 2013; Albrecht et al., 2016; Parseihian et al., 2016; Ziemer and Schultheis, 2019a), precision (Hansen et al., 2013; Parseihian et al., 2016; Ziemer and Schultheis, 2019a), accuracy (Ziemer and Schultheis, 2019a), turn arounds (Walker and Lindsay, 2006; Parseihian et al., 2016; Ziemer and Schultheis, 2019a), interruptions (Parseihian et al., 2016; Ziemer and Schultheis, 2019a), trajectory lengths (Walker and Lindsay, 2006; Ziemer and Schultheis, 2019a), trajectory entropy (Ziemer and Schultheis, 2019a), a qualitative inspection of trajectories (Lokki and Gröhn, 2005; Albrecht et al., 2016; Ziemer and Schultheis, 2019a), and training effects (Walker and Lindsay, 2006; Nagel et al., 2014; Ziemer and Schultheis, 2019a). Sometimes, navigation is evaluated in a game-like scenario in the hope of high motivation for long-term usage (Degara et al., 2013; Pires et al., 2013; Biggs et al., 2019; Ziemer and Schultheis, 2021). These kinds of evaluation have many advantages. They can show how effectively naive users can interpret and interact with the sonification, and the comparison with other sonification designs or visualization provides a benchmark. However, their major disadvantage is that the results do not clearly reveal the causes of imperfect performance. These could be of *cognitive* nature: is the information density too high? Has the given task been misunderstood? Is the participant too inexperienced in hand-ear coordination? Such cognitive aspects are largely covered by additional means, such as the subjective task load according to the NASA-task load index (TLX) (Khan and Jeon, 2018; Ziemer and Schultheis, 2019a) or the raw NASA-TLX (Black et al., 2017; Biggs et al., 2019; Ziemer and Schultheis, 2019a), the BUZZ questionnaire (Axon, 2018; Tomlinson et al., 2018; Winters and Koziej, 2020), and other subjective questionnaires, e.g., aesthetics (Vickers, 2006; Vogt, 2011; Neumann et al., 2013; Kuppanda et al., 2015), annoyance (Brewster, 2003; Vickers, 2006; Ziemer et al., 2018), clarity (Pauletto and Hunt, 2007; Vogt, 2011), comprehensibility (Vickers, 2006; Neumann et al., 2013; Yang and Hunt, 2013), distraction (Vickers, 2006; Neumann et al., 2013), informativeness (Neumann et al., 2013; Hermann et al., 2015; Khan and Jeon, 2018; Ziemer et al., 2018), intuitiveness (Pauletto and Hunt, 2007; Vogt, 2011; Kuppanda et al., 2015; Khan and Jeon, 2018), learnability/learning effort (Vogt, 2011; Neumann et al., 2013), obtrusiveness (Neumann et al., 2013), pleasantness (Pauletto and Hunt, 2007; Neumann et al., 2013; Hermann et al., 2015; Kuppanda et al., 2015), preference (Yang and Hunt, 2013), and/or utility/usefulness (Neumann et al., 2013; Khan and Jeon, 2018). These additional measures tackle the cognitive aspects of performance and provide feedback concerning the sound design of the sonification. The causes for imperfect performance could also be of *perceptual* nature: is one of the sonification dimensions not perceived as linear? Or is the perceptual resolution of one sonification dimension too low? Or do hysteresis effects along the dimension hinder the participants to interpret data? Do multiple data dimensions interfere perceptually in the sonification? These questions could be answered by applying psychoacoustic methods if the sonification is prepared for the psychoacoustic experiment and if the experiment is adopted to sonification in the execution and analysis of the results, as suggested in the PAMPAS.

### 2.2. Psychoacoustic sonification design and evaluation

*The Sonification Handbook* (Walker and Nees, 2011), p. 28 suggests that “Sonification researchers can and should, however, actively borrow from and adapt the knowledge and methods of psychoacousticians.” Even though the number of studies that applied psychoacoustic methods on sonification—magnitude estimation (Walker, 2002; Walker and Kramer, 2005) and similarity ratings (Bonebright, 2001; Fernstrom et al., 2003)—is low, there is the belief that “psychoacoustic measurements and theories can assist in the design of an auditory display” (Barrass, 1997, pp. 17–18), that psychoacoustic methods “may be the best approach to understanding how to maximize information transmission with auditory displays” (Walker and Nees, 2011, p. 28) and that direct measurements of perceptual aspects “offer a considerable advantage in the speed of data collection and are probably preferable for most applications involving the evaluation of auditory displays” (Bonebright and Flowers, 2011, p. 130).

However, this has not happened a lot until today. A reason for this may be the interdisciplinary nature of sonification. Researchers may have a background in computer science, human factors, human-computer interaction, musicology, or design, and sometimes exhibit a lack of knowledge or a misunderstanding of what psychoacoustics have to offer. For example, some sonification researchers have argued that the psychoacoustic literature neither deals with the sounds to be expected in sonification design nor aims at solving the problems that need to be assessed and overcome in sonification design (Smith, 1990; Smith et al., 1994; Barrass, 1997; Wegner and Karron, 1997; Ferguson et al., 2006; Anderson and Sanderson, 2009; Bonebright and Flowers, 2011; Bovermann et al., 2011; Vogt, 2011; Walker and Nees, 2011). This is certainly a misconception. At a first glance, psychoacoustic textbooks referred to very specific types of sound, like pure tones and Gaussian-shaped tone bursts, and signal processing, like sinusoidal amplitude and frequency modulation (Zwicker and Fastl, 1999, chap. 1; Roederer, 2009, chap. 2). However, psychoacoustic methods have also proven their validity for other types of sound, from speech over music to engineering noise and environmental sounds (Zwicker and Fastl, 1999; Leman, 2000; Roederer, 2009; Sottek, 2016; Schneider, 2018a,b).

Many sonification studies highlighted the difficulty of psychoacoustic sound synthesis (Smith, 1990; Smith et al., 1994; Wegner and Karron, 1997; Ferguson et al., 2006), because psychoacoustic models as described in Zwicker and Fastl (1999), Leman (2000), Sottek (2016) solve the forward problem, whereas psychoacoustic sound synthesis has to solve the inverse problem. Here, the forward problem is an acoustic input is given and we are required to transform it to predict the perceptual outcome. The inverse problem is: the desired perceptual outcome is given, and we need to find an acoustical input that will produce this perception. Given the complex and nonlinear transformation that our auditory system performs on the acoustic input, there is neither an analytical nor a comprehensive numerical solution to the inverse problem, except for pure tones. Here, formulas exist that transfer the physical frequency into the psychoacoustical Mel scale (Zwicker and Fastl, 1999, chap. 5; Schneider, 2018a), which describes pitch perception and the physical amplitude to the psychoacoustical phon scale (Zwicker and Fastl, 1999, chap. 8; Schneider, 2018a) and also describes loudness perception. In addition, Ferguson et al. (2006) suggested the use of psychoacoustic models in the design and evaluation phase of multidimensional sonification. They can serve to create massive lookup tables that describe the relationship between audio parameters and perceptual, auditory qualities. The benefit is that the table helps to find audio parameter combinations that produce the desired perception. The drawback is that tables can become huge, and the method can lead to jumps of audio parameters that produce audible artifacts.

However, it is true that psychoacoustic toolboxes like (Grassi and Soranzo, 2009; Soranzo and Grassi, 2014) do not work out of the box for sonification researchers. To become a powerful means for sonification evaluation, the sonification itself has to be prepared and the psychoacoustic method has to be adapted in terms of the experimental procedure and in terms of analyzing the experiment results. PAMPAS is such a means. It helps sonification researchers to assess and achieve the four requirements of multidimensional sonifications. It is described in the following two sections.

## 3. Materials and equipment

To carry out the PAMPAS on sonification design, we need a computer with an internal or external sound card, high-quality D/A-converter, amplifier, and headphones. Ideally, we can calibrate our system using an artificial ear or an artificial head that informs us about the sound pressure level to be expected at the participants' ear drums, which should lie around 70 dB.

For remote testing, an online survey platform is needed. This platform either needs to be able to play uncompressed sound files, like wav or aiff, or to render audio on the fly. More importantly, it has to be adaptive, i.e., it must be able to play a specific audio signal that depends on the user's previous actions.^1^

If our sonification design require any spatialization techniques—vector base amplitude panning (VBAP), ambisonics or wave field synthesis (Ziemer, 2020)—, we would need a respective loudspeaker setup and document the technical details on the setup.

## 4. Methods

In psychophysical terms, PAMPAS is based on a JND experiment using the maximum likelihood procedure (MLP) in a two alternative forced choice (2AFC) task. These kinds of experiments are described in detail, e.g., in Madigan and Williams (1987), Green (1990, 1993), and Grassi and Soranzo (2009).

This section starts with the aim of the presented experiment, followed by the necessary steps to prepare your data and signals and to conduct the experiment.

### 4.1. Aim

The aim of the proposed experiment is 2-fold: 1. During the sonification design stage, a single researcher or a small group of researchers and sound designers can carry out the “light” experiment to achieve a perceptually *linear* mapping. 2. After implementing a prototype, the multidimensional sonification can be evaluated in experiments with participants from the target audience to quantify

1. the *perceptual resolution* of,
2. *hysteresis effects* within, and
3. *perceptual interferences* between

each dimension and polarity. The experiment results are quantitative measures that help to assess aspects of an individual sonification design and to compare multiple sonifications.

### 4.2. Data space

**Figure 2:**
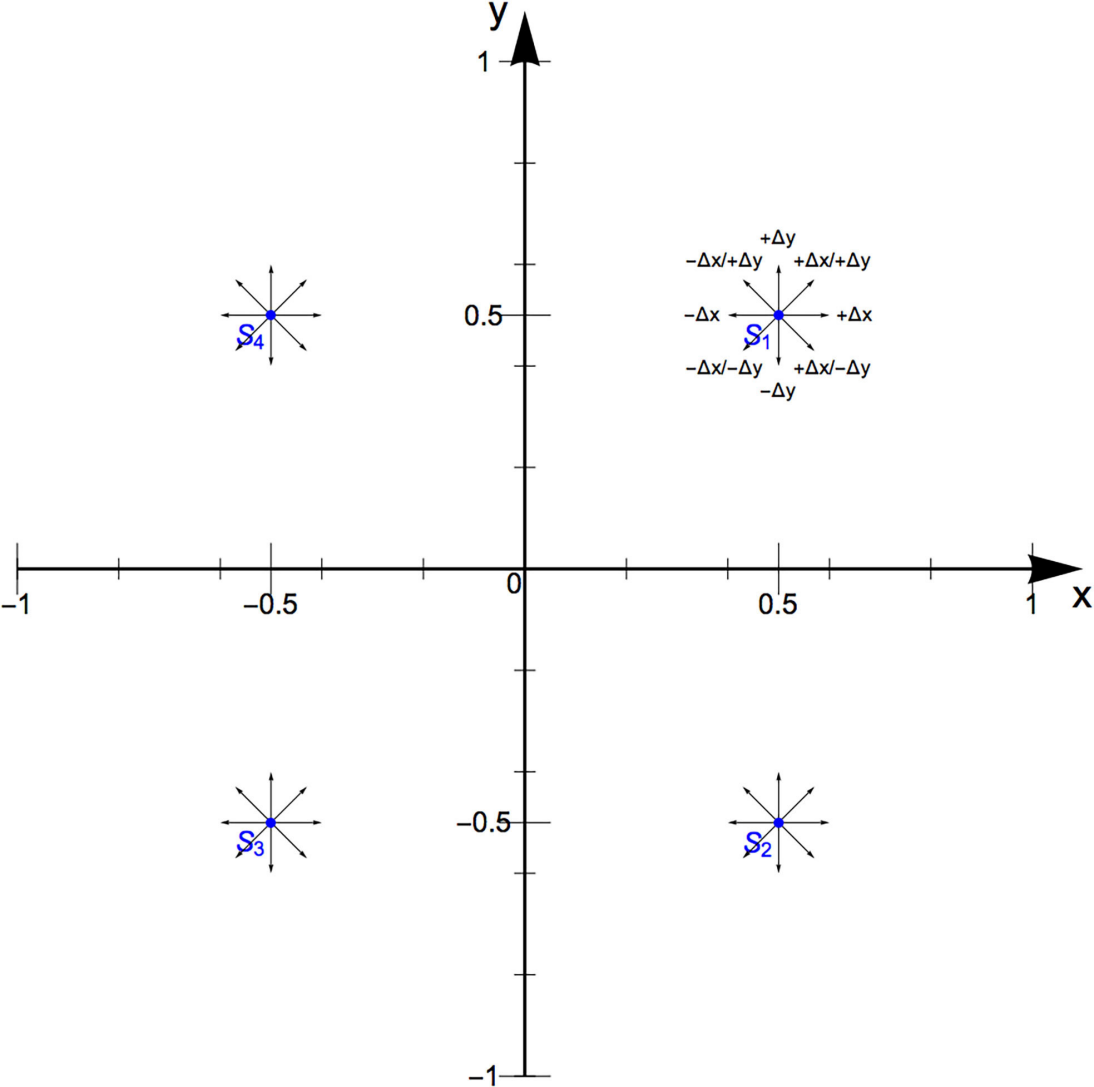
The mapping space. It has two linear dimensions that are normalized from −1 to 1. The maximum likelihood procedure (MLP) is carried out in 5 directions (small arrows) at 4 locations (*S*_I_ to *S*_IV_) to quantify the perceptual resolution, hysteresis effects, and perceptual interferences. The standards lie in the center of each quadrant.

We start with an abstract, normalized data space that has two Cartesian dimensions *x* and *y*, as illustrated in Figure 2. We refer to this as the *mapping space* that contains the *mapping data*. Each dimension has two *polarities*, a negative and a positive one, so that the dimensions range from −1 to 1. This allows us to sonify data in an interval scale. We can divide the space into 4 quadrants and name them roman I to IV. Should the sonification design be conceptualized for data in ratio scale, i.e., without a negative polarity, the data space only contains quadrant I. Naturally, a data space can also include combinations of uni- and bi-polar dimensions, i.e., quadrants I and II or quadrants I and IV.

In the center of each quadrant, we have one reference coordinate, referred to as the *standard* [*S*_I to IV_ = (±0.5, ±0.5)]. Sounds evaluated against the standard are called the *variables*. From every standard, we can either move along the positive or negative *x*-direction (±Δ*x*), the positive or negative *y*-direction (±Δ*y*), or the positive diagonal (+Δ*x*/+Δ*y*). Only these 5 motions will be tested during the experiment to keep it short.

The normalized data space is necessary in order to compare a number of evaluated sonifications, independent of their intended use case.

### 4.3. Data normalization

The abstract data space does not have to equal the data space for the intended sonification use case. If we designed our sonification to deal with values that lie well outside the range of the data space, we need to carry out a normalization.

For example, we may want to sonify if and how much the temperature *t* and the chlorine level *c* of the water in our hot tub deviate from your target temperature $t_{\text{tar}}=39^{•}$C and chlorine level *c*_tar_ = 3 ppm. Acceptable temperature ranges from $t_{\text{min}}=35^{•}$C to $t_{\text{max}}=42^{•}$C and chlorine levels from *c*_min_ = 1 ppm to *c*_max_ = 4 ppm. In this case, we normalize our sensor data in relation to our target values to conform with the data space *via*


(1) $$x=\{\begin{array}{l} 1\text{, if }t\geq t_{\text{max}}\\ \frac{t-t_{\text{tar}}}{t_{\text{max}}-t_{\text{tar}}}\text{, if }t_{\text{tar}}<t<t_{\text{max}}\\ \frac{t-t_{\text{tar}}}{t_{\text{tar}}-t_{\text{min}}}\text{, if }t_{\text{min}}<t\leq t_{\text{tar}}\\ -1\text{, if }t\leq t_{\text{min}} \end{array}$$

and


(2) $$y=\{\begin{array}{l} 1\text{, if }c\geq c_{\text{max}}\\ \frac{c-c_{\text{tar}}}{c_{\text{max}}-c_{\text{tar}}}\text{, if }c_{\text{tar}}<c<c_{\text{max}}\\ \frac{c-c_{\text{tar}}}{c_{\text{tar}}-c_{\text{min}}}\text{, if }c_{\text{min}}<c\leq c_{\text{tar}}\\ -1\text{, if }c\leq c_{\text{min}} \end{array}$$

Equations 1 and 2 are transforms from the sensed or defined *input data* to the abstract, normalized *mapping data*. The transform is also referred to as *data normalization* or *piecewise linear transfer function* (Hermann, 2002, p. 38).

Naturally, the data normalization can be preceded by additional transforms. For example, it may be meaningful to transform from the measured concentration of hydrogen ions in a liquid to the logarithmic pH value.

### 4.4. General procedure

The general procedure of a JND experiment using the 2AFC approach is simple. First, the conductor explains to the participant that a new type of informative sound is being tested. It is wise to present the sonification of discrete coordinates in ascending and descending order and visualize them on a graph similar to Figure 2, and, ideally even let the participants explore the sonification interactively themselves, using a computer mouse or any other familiar human interface device. This way, the participants can familiarize themselves with the sound, which characteristics it has, and how small or large the intensity or magnitude of each characteristic can be. To test the new type of sound, sound pairs will be presented to the participant, who has to judge which of the two has the larger intensity, magnitude, or significance of a certain characteristic, i.e., which one lies further away from the center of the coordinate origin in the respective example. They have to do this over and over, for different regions within the presented graph.

### 4.5. Signal preparation

In this section, the experiment will be explained for quadrant I. The same procedure has to be carried out for quadrants II to IV, respectively.

First, we produced the sound of the standard, i.e., we sonify coordinate (0.5, 0.5). Next, we produced the variable test sounds. For the positive *x*-direction, these are (0.5 + α, 0.5), where α goes from 0.001 to 0.1 in steps of 0.001, yielding *J* = 100 test sounds. Likewise, we produced 100 test sounds for the negative *x*-direction (0.5 − α, 0.5), for the positive *y*-direction (0.5, 0.5 + α), the negative *y*-direction (0.5, 0.5 − α) and the diagonal (0.5 + α, 0.5 + α). The sounds should last for 3 to 5 s, followed by a pause of equal length (Bonebright and Flowers, 2011, p. 114).

Note that discrete test sounds of finite duration may be different from the sound of the sonification in the intended use case, where it may be continuous. Therefore, we need to ensure that the test sounds do not provide audible cues that would not appear during the usual sonification usage. For example, we may need to ramp the signal to avoid audible clicks at the onset and offset of the sound. Imagine the *x* value was mapped to the frequency of a low frequency oscillator (LFO) that modulated the frequency of a carrier, creating a vibrato effect. Here, we must ensure that the sound of the standard and the variable start at the same phase of the LFO cycle. This way the listener's judgments will not be biased by the phase at the onset of the two sounds. Furthermore, we should choose a long ramp at the offset. This gradual fade-out will ensure that listeners do not compare the two sounds based on the phase of the LFO cycle during the note offset.

### 4.6. Psychometric functions

A psychometric function describes how likely a participant can distinguish two stimuli. This function is sampled for each individual during a 2AFC experiment and can be expressed as


(3) $$\begin{array}{l} \text{Ψ}\left(x;\alpha ,\beta ,\gamma \right)=\gamma +\left(1-\gamma \right)f\left(x;\alpha ,\beta \right)\;. \end{array}$$

Here, the chance to guess correctly lies at γ = 50%, and the function should converge to this threshold toward the lower end, the *floor*. Ideally, the *ceiling* should be 100%, even though an *attentional lapse* (Grassi and Soranzo, 2009, p. 22; Opstal, 2016, p. 220) could reduce the ceiling a bit.

The function *f*(*x*; α, β) approximates the sampled psychometric function by a sigmoid function, like the logistic function


(4) $$\begin{array}{l} f_{\text{logistic}}\left(x;\alpha ,\beta \right)=\frac{1}{1+e^{\beta \left(\alpha -x\right)}}\;, \end{array}$$

where α is the midpoint on the *x*-axis and β manipulates the slope of the curve.

### 4.7. Maximum likelihood procedure

**Figure 3:**
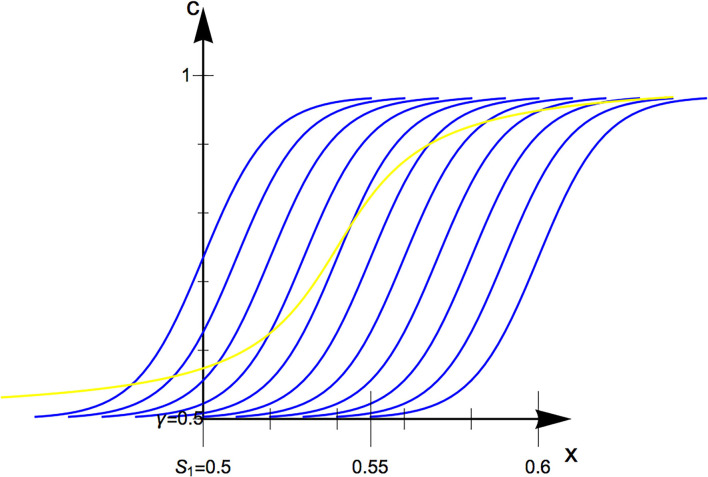
Six exemplary hypotheses (blue) and the actual psychometric function (yellow). Even though the slope is different, *H*_3_ approximates the just noticeable difference (JND) well.

Before starting the experiment, several psychometric functions are formulated, referred to as *hypotheses* *H*_*j*_. These have the same slope β and chance level γ, but different midpoints α that spread over the complete range in which the JND is assumed to lie. An example is given in Figure 3.

After every trial *n*, the likelihood of each hypothesis to equal the participant's psychometric function can be calculated as


(5) $$\begin{array}{l} L\left(H_{j}\right)=\overset{n}{\underset{i=1}{\sum }}C\mathrm{lg}H\left(x_{i}\right)+W\mathrm{lg}\left(1-H\left(x_{i}\right)\right) \end{array}$$

where *x* is the stimulus magnitude and the index *i* addresses all previous trials up to the latest trial. Here, the exponents *C* equals 1, if the participants give the correct answer, otherwise it is 0. Respectively, *W* equals 1 if the answer is wrong and 0 if the answer is correct. The hypothesis with the highest likelihood gives the best approximation of a subject's psychometric curve with the answer given from trial 1 to the *n*th trial. The calculated likelihood is recalculated every trial and becomes more accurate, so the last trial of the whole experiment returns the best estimate.

### 4.8. Stimulus selection method

It is certainly motivating to start with a clearly audible difference, like the sound of coordinate (0.57, 0.5). After each trial, the subject's most likely JND is the inverse function of his or her psychometric function at its *p*_target_, i.e.,


(6) $$\begin{array}{l} {\text{Ψ}}^{-1}\left(p_{\text{target}}\right)=\alpha_{j}-\frac{1}{\beta }\ln\left(\frac{1-\gamma }{p_{\text{target}}-\gamma }-1\right)\;. \end{array}$$

This calculated *p*_target_ will be the stimulus for the next trial. This way we ensure that most trials in the experiment are near the JND. In a 2AFC task, *p*_target_ equals 80.9%, which minimizes the variability in the estimate of the threshold (Green, 1993).

### 4.9. Conventions

Maximum likelihood procedures allow for many decisions to adapt to the given task and the specific objective of an experiment. For sonification evaluation, the main objective is to quantify

1. the perceptual resolution (JND)
2. hysteresis effects (*h*) and
3. perceptual interferences (δ) between

*x* and *y* in quadrants *S*_I to IV_.

Therefore, we suggest using the values indicated in Table 1 that allow for a quick, reliable, and comparable evaluation of two-dimensional sonification designs.

**Table 1:** Proposed values for a standardized maximum likelihood procedure (MLP) to evaluate two-dimensional sonification designs.

**Standard**	**γ**	***f*(*x*; α, β)**	**β**	**α**	***p*_target_**	***J* hypotheses**	**Trials**
(±0.5, ±0.5)	50%	$\frac{1}{1+e^{\beta \left(\alpha -x\right)}}$	100	0.001 ≤ α ≤ 0.1	80.9%	100	12

However, a premise to evaluate a multidimensional sonification with potential end-users utilizing the proposed MLP is that the sonification dimensions sound fairly linear. To assist in achieving linearity, the procedure should be carried out by the sonification designers and some colleagues with standards that equally sample the axes of the normalized data space, i.e., the tick marks in Figure 2. This “light” experiment is an aid for sonification design.

### 4.10. Demonstrations to the participants

Before we started the actual experiment, we should presented some audio examples to the participants (Bonebright and Flowers, 2011, p. 117). We suggested to sonify the coordinate (0.6, 0.6) to them as a reference, (0.601, 0.6) as a “probably inaudible difference,” (0.7, 0.6) as a “probably clearly audible difference,” and then some coordinate near our personal JND, just to give them the feel for the level of nuances they should try to achieve.

## 5. Anticipated results

At each standard *S*_I to IV_, the MLP yields 5 JNDs, namely

1. along the *x*-direction, toward *x* = 0 (i.e., JND_*S*_(−Δ*x*)) and
2. away from *x* = 0 (i.e., JND_*S*_(+Δ*x*)),
3. along the *y*-direction, toward *y* = 0 (i.e., JND_*S*_(−Δ*y*)) and
4. away from *y* = 0 (i.e., JND_*S*_(+Δ*y*)),
5. and along its diagonal (i.e., JND_*S*_(+Δ*x*, +Δ*y*).

These JNDs inform about the perceptual resolution, the presence, direction and intensity of hysteresis effects, and the degree of perceptual interference as explained below.

### 5.1. Perceptual resolution

In each quadrant, the perceptual resolution is described by two JNDs, namely the JND when increasing the absolute value of either *x* or *y*, i.e., JND_*S*_(+Δ*x*) and JND_*S*_(+Δ*y*). These directions are indicated as the orthogonal, green arrows in Figure 2.

For example, the JND_*S*_I__(+Δ*x*) reveals the perceptual resolution of the *x*-dimension in quadrant I. Ideally, these JNDs are very small, indicating a high perceptual resolution of the respective dimension. In the proposed method, the smallest possible value is 0.001, which would indicate that 1/0.001 = 1000 steps can be distinguished by listeners in quadrant I, given that the parameter mapping along the respective dimension is truly linear from *x* = 0 to *x* = 1. The highest possible JND is 0.1, which would indicate that 1/0.1 = 10 steps can be distinguished by listeners. This score is very low. Such a sonification is not suitable for the presentation of a continuous axis in interval or ratio scale, but rather for ordinal or nominal data.

The perceptual resolution should be reported as a table, as demonstrated in Table 2.

**Table 2:** The perceptual resolution of each dimension in each quadrant.

	**I**	**II**	**III**	**IV**
*x*	JND_*S*_I__(+Δ*x*)	JND_*S*_II__(+Δ*x*)	JND_*S*_III__(+Δ*x*)	JND_*S*_IV__(+Δ*x*)
*y*	JND_*S*_I__(+Δ*y*)	JND_*S*_II__(+Δ*y*)	JND_*S*_III__(+Δ*y*)	JND_*S*_IV__(+Δ*y*)

Preceding the sonification evaluation experiment, sonification designers should ensure linearity of the axis by measuring JND(+Δ*x*) at each tick mark along the *x*-axis and JND(+Δ*y*) at each tick mark along the *y*-axis in a “light” self-experiment. Here, the JND at every tick mark of each axis polarity should be equal. If this is not the case, the parameter mapping function needs to be adjusted.

### 5.2. Hysteresis effects

In each quadrant, the ratio between the JND along the positive and along the negative direction of a dimension informs us about hysteresis effects. Ideally, JND_*S*_(−Δ*x*) equals JND_*S*_(+Δ*x*), meaning that no hysteresis effects exist along *x* in the respective quadrant. If either JND is greater, it implies that larger changes along that direction are necessary in order to be audible. As a fairly linear mapping is a prerequisite for the listening experiments, the ratio between the two JNDs indicates the strength and direction of a hysteresis effect, calculated as


(7) $$\begin{array}{l} h_{S}\left(x\right)=0.5\mathrm{lg}\frac{{\text{JND}}_{S}\left(-\Delta x\right)}{{\text{JND}}_{S}\left(+\Delta x\right)}\;. \end{array}$$

The fraction in Equation 7 yields a positive value that lies between 0.001/0.1 = 10^−2^ and 0.1/0.001 = 10^2^, so the score *h*_*S*_(*x*) is normalized to values between −1 and 1. Here, positive values mean that when approaching the coordinate origin, larger steps are necessary in order to be audible, compared to the situation in which you move away from the coordinate origin. In other words, positive values indicate that the perceptual resolution in the direction of the coordinate origin is higher compared to the other direction along the respective dimension. The larger the absolute value of *h*_*S*_(*x*), the stronger this hysteresis effect. Naturally, a respective *h*_*S*_(*y*) quantifies the hysteresis effects along the *y*-dimension.

The hysteresis scores should be reported as a table for each quadrant and direction, as demonstrated in Table 3.

**Table 3:** The hysteresis effects of each dimension in each quadrant.

	**I**	**II**	**III**	**IV**
*x*	*h*_*S*_I__(*x*)	*h*_*S*_II__(*x*)	*h*_*S*_III__(*x*)	*h*_*S*_IV__(*x*)
*y*	*h*_*S*_I__(*y*)	*h*_*S*_II__(*y*)	*h*_*S*_III__(*y*)	*h*_*S*_IV__(*y*)

Whether hysteresis effects are acceptable or problematic depends on the intended use case.

### 5.3. Perceptual interference

The diagonal JND, i.e., JND_*S*_(+Δ*x*, +Δ*y*), is an indicator of perceptual interference, especially in relation to the JNDs of the single dimensions JND_*S*_(+Δ*x*) and JND_*S*_(Δ*y*). These are indicated as green arrows in Figure 2.

First, we define the minimum JND_*S*_min__ as the smaller value of JND_*S*_(+Δ*x*) and JND_*S*_(+Δ*y*), i.e.,


(8) $$\begin{array}{l} {\text{JND}}_{S_{\text{min}}}=\text{min}\left({\text{JND}}_{S}\left(+\Delta x\right),{\text{JND}}_{S}\left(+\Delta y\right)\right) \end{array}$$

and the maximum JND_*S*_max__ as the larger one, i.e.,


(9) $$\begin{array}{l} {\text{JND}}_{S_{\text{max}}}=\text{max}\left({\text{JND}}_{S}\left(+\Delta x\right),{\text{JND}}_{S}\left(+\Delta y\right)\right)\;. \end{array}$$

Their ratio is our threshold *T* defined as


(10) $$\begin{array}{l} T_{S}=0.5\mathrm{lg}\frac{{\text{JND}}_{S_{\text{max}}}}{{\text{JND}}_{S_{\text{min}}}}\;. \end{array}$$

Equation 10 is similar to our hysteresis definition, Equation 7. But as min ≤ max, *T*_*S*_ can only take values from 0 to 1.

The degree of perceptual interference δ is defined as


(11) $$\begin{array}{l} \delta_{S}=0.5\mathrm{lg}\frac{{\text{JND}}_{S}\left(+\Delta x,+\Delta y\right)}{{\text{JND}}_{S_{\text{min}}}}\;. \end{array}$$

Here, the score δ_*S*_ could theoretically take values from −1 to 1. Qualitatively, we can distinguish four cases of perceptual interference *Q*(δ), namely


(12) $$Q(\delta )=\{\begin{array}{l} \text{positive interference, if }\delta_{S}<0\\ \text{no interference, if }\delta_{S}=0\\ \text{usual interference, if }0<\delta_{S}\leq T_{S}\\ \text{negative interference, if }\delta_{S}>T_{S} \end{array}.$$

Positive interference is a rare case in which changes along one dimension improve the user's precision in detecting changes along the second dimension. No interference implies that the two dimensions are orthogonal, which is also very rare not only in audition, but also in psychology in general. The usual interference implies that the JND along the diagonal lies somewhere between the JND of the single dimensions. If the JND of the diagonal is even larger than the larger of the two single-dimension JNDs, we refer to that as negative interference.

The quantitative and qualitative interference of all quadrants should be reported in a table, as shown in Table 4.

**Table 4:** The perceptual interference in each quadrant.

	**δ**	***Q***
*S*_I_	δ_*S*_I__	*Q*(δ_*S*_I__)
*S*_II_	δ_*S*_II__	*Q*(δ_*S*_II__)
*S*_III_	δ_*S*_III__	*Q*(δ_*S*_III__)
*S*_IV_	δ_*S*_IV__	*Q*(δ_*S*_IV__)

## 6. Discussion

In this paper, we gave an overview of design and evaluation methods of sonification and the ongoing debate on the potentials and limitations of psychoacoustic methods to assist in sonification research.

A PsychoAcoustical Method for the Perceptual Analysis of multidimensional Sonification utilizes an MLP in a JND experiment to apply on multidimensional sonifications.

The proposed MLP for the evaluation of two-dimensional sonification designs fulfills the three main quality criteria of scientific test:

1. It is objective, as the procedure and the measures are standardized, and even the qualitative categories are derived from quantitative thresholds.
2. It is reliable, as has been demonstrated by numerous comparative studies (Pentland, 1980; Shelton et al., 1982; Madigan and Williams, 1987), and also has a high test-retest reliability.
3. Its validity is high for passive data sonification because participants of the experiment carry out a task related to the typical usage of passive sonification.

Applying the proposed experiment on a two-dimensional sonification of a normalized data space yields quantitative and qualitative results that allow the evaluation of a single sonification design. As the method is standardized, the experiment is repeatable and results can be compared between multiple studies.

PsychoAcoustic Method for the Perceptual Analysis of multidimensional Sonification has many advantages:

1. It can aid in achieving perceptual linearity during the sonification design stage (by using the “light” experiment). Here, PAMPAS “light” helps identify by how much (Δβ) an audio parameter β of any sonification has to be increased for different values of β to be just noticeable. Perceptual linearity is important for a proper understanding of data trends and relations.
2. It directly measures the perceptual resolution of each axis of a sonification, given by the JND. The JND is a perceptual limitation that can hardly be overcome through better instructions, training, or concentration.
3. It quantifies the degree of perceptual interference between the two axes of a sonification, given by δ. Perceptual interference can obscure the data. In Cartesian coordinates, for example, the distance and angle of a coordinate can only be derived from the *x*- and the *y*-coordinate if both axes are orthogonal and perceptually independent.
4. It quantifies the amount and the direction of hysteresis effects of each sonification dimension, given by *h*. Depending on the intended use case, hysteresis effects in sonification may be acceptable and could be counter-balanced, if the amount and direction of hysteresis effects are known.
5. It provides comparability between various sonification designs, even if the experiments are carried out by different research groups. This can save a lot of experimental time.

This way PAMPAS is a powerful means to evaluate the perceptual aspects of multidimensional sonification directly. As mentioned above, evaluating sonifications by letting participants solve a task using one or multiple sonifications and then visualizing, which is a common practice, to reveal performance of users. Additional measures, like NASA-TLX, BUZZ, and other questionnaires reveal cognitive aspects that may contribute to the measured performance. What has been missing was measures to reveal perceptual aspects that may contribute to the measured performance. These are delivered by PAMPAS. Consequently, PAMPAS does not replace other user experiments but complements them.

Note that the proposed method has several limitations, too.

1. The participants in the experiment are attentively focused on judging the sound. This may also be the case for the intended use case of the sonification. But it may also be the actual use case involving shared attention, which is likely to increase the JND and the amount of interference, due to cognitive aspects (Anderson and Sanderson, 2009).
2. The participants in the experiment do not interact with the sound. This may be the case in a data monitoring task. But it may also be that the user interacts with the data and the sound. It can reduce the JND and also hysteresis effects if changes in sound are meaningfully related to (inter-)action (cf. Ziemer and Schultheis, 2018). But it may also increase the JND if the sound does not behave in an expected way.
3. The experiment is unimodal. This may be the same for the intended use case. But in other use cases, the user may also receive cues through other sensory systems, such as vision and touch. Multi-modal interference, such as the McGurck effect and the ventriloquist effect (Ziemer, 2020, chap. 4) between vision and audition, may affect the JND, interferences, and hystereses.
4. The experiment gives participants sufficient time to listen closely to the sonification. In a real use case, the sound may change quickly. The experiment does not reveal the temporal resolution of the multidimensional sonification, i.e., it is possible that the JND increases when the data fluctuate quickly.
5. The participants in the experiment are new to the stimuli. This may certainly be the case for first-time users of the sonification. However, users may familiarize themselves with the sound and thereby learn what to listen for, i.e., which sound aspect to focus on, potentially reducing the JND and interferences. The experiment does not reveal such kinds of training effects, which should be addressed through longitudinal studies (Walker and Lindsay, 2003; Ziemer and Schultheis, 2021).
6. The task in the described experiment is abstract. In an actual implementation of the sonification, the sound has a certain context and a relationship to a certain task or aspect related to the sonified data, which may affect sound perception due to cognitive effects.
7. Observations on interferences between two dimensions only focus on small, equal changes of both dimensions, i.e., the diagonal. It is not clear how these interferences transfer to larger changes and to directions other than the diagonal.
8. The method is limited to aspects of auditory perception. It does not include perception from other senses, such as vision or touch. It does not consider cognitive aspects, like fatigue, misunderstanding of a task, or inability to judge the experiment stimuli independent from “unavoidable physiological noises (heartbeat, pulse, breathing, blood rushing through vessels, stomach gurgles, etc.)” (Gelfand, 2010, p. 158). It also does not investigate technical aspects of the user interface. Consequently, it should supplement the well-established methods of sonification design and evaluation, like navigation tasks, NASA TLX and subjective questionnaires.
9. The method is only described for two-dimensional sonifications as it is typical to test for orthogonality between two dimensions at a time (Neuhoff, 2004). However, to evaluate a three-dimensional sonification, the experiment can be conducted repeatedly, using *x*-*y*, *x*-*z*, and *y*-*z* axes (Ziemer and Schultheis, 2019c).

Despite these shortcomings, the proposed method is a good starting point for a perceptually-meaningful sound evaluation during the sonification design stage and in the assessment phase. It should be followed by additional experiments that address the open questions.

Note that many more psychoacoustic methods exist and may be suitable to aid sonification design and evaluation if properly adapted to the aims and signals of sonification. For example, consulting psychoacoustic models (Aures, 1985a,b; Zwicker and Fastl, 1999), and especially those designed for transient sounds like (Leman, 2000; Sottek, 2016), could give a reasonable prediction of perceptual linearity, orthogonality, and *psychoacoustic annoyance* (Zwicker and Fastl, 1999, chap. 16).

## Data availability statement

The original contributions presented in the study are included in the article/supplementary material, further inquiries can be directed to the corresponding author.

## Author contributions

TZ has written the initial draft of the manuscript. HS has reviewed and complimented the manuscript. Both authors have conceptualized the described method and tested the procedure.

## Conflict of interest

The authors declare that the research was conducted in the absence of any commercial or financial relationships that could be construed as a potential conflict of interest.

## Publisher's note

All claims expressed in this article are solely those of the authors and do not necessarily represent those of their affiliated organizations, or those of the publisher, the editors and the reviewers. Any product that may be evaluated in this article, or claim that may be made by its manufacturer, is not guaranteed or endorsed by the publisher.

## Nomenclature

I to IV, Quadrants 1 to 4 in a Cartesian coordinate system; JND, Just Noticeable Difference; LFO, Low Frequency Oscillator; MLP, Maximum Likelihood Procedure; NASA-TLX, NASA Task Load Index; PAMPAS, PsychoAcoustic Method for the Perceptual Analysis of multidimensional Sonification.

## Footnotes

^1^Amongst others, MATLAB Online/MATLAB Web App Server fulfills these demands. It is one of the platforms suggested by the Task Force on Remote Testing by the Technical Committee on Psychological and Physiological Acoustics of the Acoustical Society of America: https://www.spatialhearing.org/remotetesting/Main/HomePage and an MLP toolbox for MATLAB is freely available under https://dpg.unipd.it/en/mlp/mlp-toolbox.

## References

[B1] Albrecht R. Väänänen R. Lokki T. (2016). Guided by music: pedestrian and cyclist navigation with route and beacon guidance. Pers. Ubiquit. Comput. 20, 121–145. 10.1007/s00779-016-0906-z

[B2] Anderson J. E. Sanderson P. (2009). Sonification design for complex work domains: dimensions and distractors. J. Exp. Psychol. Appl. 15, 183–198. 10.1037/a0016329 19751070

[B3] Asendorf M. Kienzle M. Ringe R. Ahmadi F. Bhowmik D. Chen J. . (2021). Tiltification—An accessible app to popularize sonification, in The 26th International Conference on Auditory Display (ICAD 2021), 184–191. Available online at: https://smartech.gatech.edu/handle/1853/66331?show=full

[B4] Aures W. (1985a). Berechnungsverfahren für den sensorischen Wohlklang beliebiger Schallsignale (a model for calculating the sensory euphony of various sounds). Acustica 59, 130–141.

[B5] Aures W. (1985b). Ein berechnungsverfahren der rauhigkeit (a procedure for calculating auditory roughness). Acta Acust United Ac 58, 268–281.

[B6] Axon L. (2018). Sonification for network-security monitoring (Ph.D. thesis). University of Oxford.

[B7] Barrass S. (1997). Auditory Information Design (Ph.D. thesis). Australian National University, Canberra.

[B8] Barrass S. Kramer G. (1999). Using sonification. Multimedia Syst. 7, 23–31. 10.1007/s005300050108

[B9] Biggs B. Coughlan J. M. Coppin P. (2019). Design and evaluation of an audio game-inspired auditory map interface, in 25th International Conference on Auditory Display (ICAD 2019) (Newcastle upon Tyne), 20–27. 10.21785/icad2019.051 PMC7015068 32051791

[B10] Black D. Hettig J. Luz M. Hansen C. Kikinis R. Hahn H. (2017). Auditory feedback to support image-guided medical needle placement. Int. J. Comput. Assist. Radiol. Surg. 12, 1655–1663. 10.1007/s11548-017-1537-1 28213646 PMC5561528

[B11] Bonebright T. L. (2001). Perceptual structure of everyday sounds: A multidimensional scaling approach, in Proceedings 7th International Conference on Auditory Display (ICAD2001) (Espoo), 73–78.

[B12] Bonebright T. L. Flowers J. H. (2011). Evaluation of auditory display, in The Sonification Handbook, eds Hermann T. Hunt A. Neuhoff J. G. (Berlin: COST and Logos), 111–144.

[B13] Bovermann T. Rohrhuber J. de Campo A. (2011). Laboratory methods for experimental sonification, in The Sonification Handbook, Chapter 10, eds Hermann T. Hunter A. Neuhoff J. G. (Berlin: COST and Logos), 237–272.

[B14] Brewster S. (2003). Nonspeech auditory output, in The Human-Computer Interaction Handbook, eds Jacko J. A. Sears A. (London: Lawrence Erlbaum Associates), 220–239.

[B15] Canévet G. Scharf B. (1990). The loudness of sounds that increase and decrease continuously in level. J. Acoust. Soc. Am. 88, 2136–2142. 10.1121/1.400110 2269729

[B16] Chambers C. Pressnitzer D. (2014). Perceptual hysteresis in the judgment of auditory pitch shift. Atten. Percept. Psychophys. 76, 1271–1279. 10.3758/s13414-014-0676-5 24874257

[B17] Degara N. Kuppanda T. Nagel F. (2013). The walking game: a framework for evaluating sonification methods in blind navigation, in Proceedings of the 4th Interactive Sonification Workshop (ISon2013) (Erlangen), 52–57.

[B18] Fechner G. T. (1860). Elemente der Psychophysik. Leipzig: Breitkopf und Härtel

[B19] Ferguson S. Cabrera D. Beilharz K. Song H.-J. (2006). Using psychoacoustical models for information sonification, in 12th International Conference on Auditory Display (London), 113–120.

[B20] Fernstrom M. Brazil E. Ottaviani L. (2003). A new experimental technique for gathering similarity ratings for sounds, in Proceedings of 9th International Conference on Auditory Display (ICAD2003) (Boston, MA), 238–242.

[B21] Feron F.-X. Frissen I. Guastavino C. (2009). Upper limits of auditory motion perception: the case of rotating sounds, in Proceedings of the 15th International Conference on Auditory Display (ICAD2009 (Copenhagen), 6.

[B22] Fitch W. Kramer G. (1993). Sonifying the body electric: superiority of an auditory over a visual display in a complex multivariate system, in Auditory Display: Sonification, Audification and Auditory Interfaces, volume XVIII of SFI Studies in the Sciences of Complexity, ed Kramer G. (Reading, MA: Perseus Publishing), 307–325.

[B23] Gelfand S. A. (2010). Hearing, in An Introduction to Psychological and Physiological Acoustics, 5th Edn (London: Informa).

[B24] Grassi M. Soranzo A. (2009). MLP: a MATLAB toolbox for rapid and reliable auditory threshold estimations. Behav. Res. Methods 41, 20–28. 10.3758/BRM.41.1.20 19182120

[B25] Green D. M. (1990). Stimulus selection in adaptive psychophysical procedures. J. Acoust. Soc. Am. 87, 2662–2674. 10.1121/1.399058 2373801

[B26] Green D. M. (1993). A maximum-likelihood method for estimating thresholds in a yes-no task. J. Acoust. Soc. Am. 93, 2096–2105. 10.1121/1.406696 8473622

[B27] Greenwood D. D. (1997). The Mel Scale's disqualifying bias and a consistency of pitch-difference equisections in 1956 with equal cochlear distances and equal frequency ratios. Hear. Res. 103, 199–224. 10.1016/S0378-5955(96)00175-X 9007585

[B28] Greindl A. Heidegger P. Groß-Vogt K. Weger M. (2020). Expergefactor: Sonic interaction design for an alarm clock app, in Proceedings of the 15th International Audio Mostly Conference (AM?20) (Graz), 4.

[B29] Hansen C. Black D. Lange C. Rieber F. Lamad,é W. Donati M. . (2013). Auditory support for resection guidance in navigated liver surgery. Int. J. Med. Robot. Comput. Assist. Surgery 9, 36–43. 10.1002/rcs.1466 23192891

[B30] Hermann T. (2002). Sonification for Exploratory Data Analysis (Ph.D. thesis). Bielefeld University, Bielefeld.

[B31] Hermann T. (2008). Taxonomy and definitions for sonification and auditory display, in 14th International Conference on Auditory Display (Paris), 8.

[B32] Hermann T. (2018). Wave space sonification, in 24th International Conference on Auditory Display (ICAD2018) (Houghton, MI), 49–56.

[B33] Hermann T. (2021). Sonification-a Definition. Available online at: https://sonification.de/son/definition/ (accessed March 19, 2021).

[B34] Hermann T. Hildebrandt T. Langeslag P. Rinderle-Ma S. (2015). Optimizing aesthetics and precision in sonification for peripheral process-monitoring, in Proceedings of the 21st International Conference on Auditory Display (ICAD2015) (Graz), 317–318.

[B35] Ibrahim A. A. A. Yassin F. M. Sura S. Andrias R. M. (2011). Overview of design issues and evaluation of sonification applications, in Proceedings of International Conference on User Science and Engineering (i-USEr) (Selangor), 77–82.

[B36] Khan R. A. Jeon M. (2018). “musical exercise” for people with visual impairments: a preliminary study with the blindfolded, in 24th International Conference on Auditory Display (ICAD2018) (Houghton, MI), 204–211.

[B37] Komatsu T. Yamada S. (2016). Can monaural auditory displays convey directional information to users? in Proceedings of the 38th Annual Conference of the Cognitive Science Society, eds Papafragou A. Grodner D. Mirman D. Trueswell J. C. (Austin, TX: Cognitive Science Society), 930–935.

[B38] Kuppanda T. Degara N. Worrall D. Thoshkahna B. Müller M. (2015). Virtual reality platform for sonification evaluation, in The 21ts International Conference on Auditory Display (ICAD) (Graz), 117–124.

[B39] Leman M. (2000). Visualization and calculation of the roughness of acoustical musical signals using the synchronization index model (SIM), in Proceedings of the Third International Conference on Digital Audio Effects (DAFx-00) (Verona), 6.

[B40] Lokki T. Gröhn M. (2005). Navigation with auditory cues in a virtual environment. IEEE Multimedia 12, 80–86. 10.1109/MMUL.2005.33

[B41] Madigan R. Williams D. (1987). Maximum-likelihood psychometric procedures in two-alternative forced-choice: evaluation and recommendations. Percept. Psychophys. 42, 240–249. 10.3758/BF03203075 3671049

[B42] Martens W. L. (2002). Rapid psychophysical calibration using bisection scaling for individualized control of source elevation in auditory display, in Proceedings of the 8th International Conference on Auditory Display (ICAD2002) (Kyoto), 8.

[B43] Munzner T. (2014). Visualization Analysis and Design. Boca Raton, FL: CRC Press.

[B44] Nagel F. Stöter F.-R. Degara N. Balke S. Worrall D. (2014). Fast and accurate guidance-response times to navigational sounds, in ICAD (New York, NY), 5.

[B45] Nees M. A. Walker B. N. (2009). Auditory interfaces and sonification, in The Universal Access Handbook, ed Stephanidis (New York, NY: CRC Press), 507–522.

[B46] Neuhoff J. G. (1998). Perceptual bias for rising tones. Nature 395, 123–124. 10.1038/25862 9744266

[B47] Neuhoff J. G. (2004). Interacting perceptual dimensions, in Ecological Psychoacoustics, Chapter 10, ed Neuhoff J. G. (San Diego, CA: Elsevier), 249–269.

[B48] Neuhoff J. G. (2019). Is sonification doomed to fail?, in Proceedings of 25th International Conference on Auditory Display (Newcastle upon Tyne), 3.

[B49] Neuhoff J. G. Kramer G. (2000). Sonification and the interaction of perceptual dimensions: can the data get lost in the map?, in Proceedings of 6th International Conference on Auditory Display (ICAD2000) (Atlanta, GA), 6.

[B50] Neuhoff J. G. Kramer G. Wayand J. (2002). Pitch and loudness interact in auditory displays: Can the data get lost in the map? J. Exp. Psychol. Appl. 8, 17–25. 10.1037/1076-898X.8.1.17 12009173

[B51] Neumann A. Hermann T. Tünnermann R. (2013). Interactive sonification to support joint attention in augmented reality-based cooperation, in Proceedings of ISon 2013, 4th Interactive Sonification Workshop (Erlangen), 58–64.

[B52] Olivetti Belardinelli M. Federici S. Delogu F. Palmiero M. (2009). Sonification of spatial information: audio-tactile exploration strategies by normal and blind subjects, in Universal Access in Human-Computer Interaction. Intelligent and Ubiquitous Interaction Environments: 5th International Conference, UAHCI 2009, Held as Part of HCI International 2009, San Diego, CA, USA, July 19-24, 2009. Proceedings, Part II, ed Stephanidis C. (Berlin; Heidelberg: Springer), 557–563.

[B53] Opstal J. (2016). The Auditory System and Human Sound-Localization Behavior. San Diego, CA: Academic Press.

[B54] Parseihian G. Gondre C. Aramaki M. Ystad S. Kronland-Martinet R. (2016). Comparison and evaluation of sonification strategies for guidance tasks. IEEE Trans. Multimedia 18, 674–686. 10.1109/TMM.2016.2531978

[B55] Pauletto S. Hunt A. (2007). Interacting with sonifications: an evaluation, in Proceedings of 13th International Conference on Auditory Display (ICAD2007) (Montréal, QC), 519–525.

[B56] Pentland A. (1980). Maximum likelihood estimation: the best PEST. Percept. Psychophys. 28, 377–379. 10.3758/BF03204398 7465322

[B57] Pires D. Furtado B. Carregã T. Reis L. Pereira L. L. Craveirinha R. . (2013). The blindfold soundscape game: A case for participation-centered gameplay experience design and evaluation, in Proceedings of the 8th Audio Mostly Conference, AM '13 (New York, NY: Association for Computing Machinery), 1–7.

[B58] Roederer J. G. (2009). The physics and psychophysics of music, in An Introduction, 4th Edn (New York, NY: Springer).

[B59] Scaletti C. (2018). Sonification ≠ music, in The Oxford Handbook of Algorithmic Music, eds Dean R. T. McLean A. (New York, NY: Oxford University Press), 363–385.

[B60] Scheminzky F. (1943). Die Welt des Schalls, Salzburg: Das Bergland-Buch.

[B61] Schneider A. (2018a). Fundamentals, in Springer Handbook of Systematic Musicology, ed Bader R. (Berlin; Heidelberg: Springer), 559–603.

[B62] Schneider A. (2018b). Pitch and pitch perception, in Springer Handbook of Systematic Musicology, ed Bader R. (Berlin; Heidelberg: Springer Berlin Heidelberg), 605–685.

[B63] Seiça M. Roque L. Martins P. Cardoso F. A. (2020). Contrasts and similarities between two audio research communities in evaluating auditory artefacts, in Proceedings of the 15th International Conference on Audio Mostly (New York: NY), 183–190.

[B64] Shelton B. R. Picardi M. C. Green D. M. (1982). Comparison of three adaptive psychophysical procedures. J. Acoust. Soc. Am. 71, 1527–1533. 10.1121/1.3878062798028

[B65] Smith S. (1990). Representing data with sound, in Proceedings of IEEE Visualization (Piscataway, NJ: IEEE).

[B66] Smith S. Pickett R. M. Williams M. G. (1994). Environments for exploring auditory representations of multidimensional data, in Auditory Display: Sonification, Audification, and Auditory Interfaces, ed Kramer G. (Reading, MA: Addison-Wesley), 167–183.

[B67] Soranzo A. Grassi M. (2014). PSYCHOACOUSTICS: a comprehensive MATLAB toolbox for auditory testing. Front. Psychol. 5, 712. 10.3389/fpsyg.2014.00712 25101013 PMC4104800

[B68] Sottek R. (2016). A hearing model approach to time-varying loudness. Acta Acust. United Ac. 102, 725–744. 10.3813/AAA.91898926122652

[B69] Stevens S. S. Volkmann J. (1940). The relation of pitch to frequency: a revised scale. Am. J. Psychol. 53, 329–353. 10.2307/1417526

[B70] Supper A. (2012). The search for the "killer application": drawing the boundaries around the Sonification of scientific data, in The Oxford Handbook of Sound Studies, eds Pinch T. Bijsterveld K. (New York, NY: Oxford University Press), 249.

[B71] Tomlinson B. J. Noah B. E. Walker B. N. (2018). BUZZ: an auditory interface user experience scale, in Extended Abstracts of the 2018 CHI Conference on Human Factors in Computing Systems, CHI EA '18 (New York, NY: Association for Computing Machinery), 1–6.

[B72] Vickers P. (2006). Lemma 4: Haptic input + auditory display = musical instrument? in Haptic and Audio Interaction Design, eds McGookin D. Brewster S. (Berlin; Heidelberg: Springer Berlin Heidelberg), 56–67.

[B73] Vogt K. (2011). A quantitative evaluation approach to sonifications, in Proceedings of 17th International Conference on Auditory Display (ICAD2011) (Hungary: Budapest), 8.

[B74] Walker B. N. (2002). Magnitude estimation of conceptual data dimensions for use in sonification. J. Exp. Psychol. Appl. 8, 211–221. 10.1037/1076-898X.8.4.211 12570096

[B75] Walker B. N. Kramer G. (2005). Mappings and metaphors in auditory displays: an experimental assessment. ACM Trans. Appl. Percept. 2, 407–412. 10.1145/1101530.1101534

[B76] Walker B. N. Lindsay J. (2003). Effect of beacon sounds on navigation performance in a virtual reality environment, in Proceedings of the 2003 International Conference on Auditory Display (ICAD2003) (Boston, MA), 204–207.

[B77] Walker B. N. Lindsay J. (2006). Navigation performance with a virtual auditory display: effects of beacon sound, capture radius, and practice. Hum. Factors 48, 265–278. 10.1518/001872006777724507 16884048

[B78] Walker B. N. Nees M. A. (2011). Theory of sonification, in The Sonification Handbook, Chapter 2, eds Hermann T. Hunt A. Neuhoff J. G. (Berlin: COST and Logos), 9–39.

[B79] Watson M. Sanderson P. (2004). Sonification supports eyes-free respiratory monitoring and task time-sharing. Hum. Factors 46, 497–517. 10.1518/hfes.46.3.497.50401 15573548

[B80] Wegner C. M. Karron D. (1997). Surgical navigation using audio feedback, in Medicine Meets Virtual Reality: Global Healthcare Grid, volume 39 of Studies in Health Technology and Informatics, eds Morgan K. Hoffman H. Stredney D. Weghorst S. (Ohmsha; Washington, DC: IOS Press), 450–458. 10173065

[B81] Winters R. M. Koziej S. (2020). An auditory interface for realtime brainwave similarity in dyads, in Proceedings of the 15th International Conference on Audio Mostly (Graz: Association for Computing Machinery), 261–264.

[B82] Worrall D. (2014). Can micro-gestural inflections be used to improve the soniculatory effectiveness of parameter mapping sonifications? Organ. Sound 19, 52–59. 10.1017/S135577181300040X

[B83] Worrall D. (2019). Sonification Design. Cham: Springer.

[B84] Yang J. Hunt A. (2013). Sonic trainer: real-time sonification of muscular activity and limb positions in general physical exercise, in Proceedings of ISon 2013, 4th Interactive Sonification Workshop (Erlangen), 44–51.

[B85] Yeung E. S. (1980). Pattern recognition by audio representation of multivariate analytical data. Anal. Chem. 52, 1120–1123. 10.1021/ac50057a028

[B86] Ziemer T. (2020). Psychoacousticmusic Sound Field Synthesis, Volume 7 of Current Research in Systematic Musicology. Cham: Springer.

[B87] Ziemer T. Nuchprayoon N. Schultheis H. (2020). Psychoacoustic sonification as user interface for human-machine interaction. Int. J. Inform. Soc. 12, 3–16. 10.13140/RG.2.2.14342.11848

[B88] Ziemer T. Schultheis H. (2018). A psychoacoustic auditory display for navigation, in 24th International Conference on Auditory Displays (ICAD2018) (Houghton, MI), 136–144.

[B89] Ziemer T. Schultheis H. (2019a). Psychoacoustic auditory display for navigation: an auditory assistance system for spatial orientation tasks. J. Multimodal User Interfaces 13, 205–218. 10.1007/s12193-018-0282-2

[B90] Ziemer T. Schultheis H. (2019b). Psychoacoustical signal processing for three-dimensional sonification, in 25th International Conference on Auditory Displays (ICAD2019) (Newcastle), 277–284.

[B91] Ziemer T. Schultheis H. (2019c). Three orthogonal dimensions for psychoacoustic sonification. arXiv [Preprint]. arXiv:1912.00766. 10.48550/arXiv.1912.00766

[B92] Ziemer T. Schultheis H. (2020). Linearity, orthogonality, and resolution of psychoacoustic sonification for multidimensional data. J. Acoust. Soc. Am. 148, 2786–2786. 10.1121/1.5147752

[B93] Ziemer T. Schultheis H. (2021). The CURAT sonification game: gamification for remote sonification evaluation, in The 26th International Conference on Auditory Display (ICAD 2021), 233–240. Available online at: https://smartech.gatech.edu/handle/1853/66332?show=full

[B94] Ziemer T. Schultheis H. Black D. Kikinis R. (2018). Psychoacoustical interactive sonification for short range navigation. Acta Acustica United Acust. 104, 1075–1093. 10.3813/AAA.919273

[B95] Zwicker E. Fastl H. (1999). Psychoacoustics. Facts and Models, 2nd Edn. Berlin; Heidelberg: Springer.

